# New and Ready Mode of Producing Anæsthesia

**Published:** 1866-06

**Authors:** 


### NEW AND READY MODE OF PRODUCING ANÆSTHESIA.

Dr. B. W. Richardson has been for some years engaged in researches for the production of local anæsthesia. Snow maintained that all narcotics produced anæsthesia by the process of arresting oxidation. Dr. R. has come to the conclusion that arrest of oxidation means arrest of motion, and that anæsthesia means the temporary death of a part, *i.e.*, inertia in the molecules of the part. This led him to the conclusion that Dr. Arnott's plan of using extreme cold was the first true step in the progress of discovery, and that if it could be made easier of application, and at the same time could be combined with the use of a narcotic fluid an important advance in therapeutics would necessarily follow. Dr. R. has been for four years engaged in experimenting with a view of demonstrating this. Finally he has devised an apparatus consisting "simply of a graduated bottle for holding ether; through a perforated cork a double tube is inserted, one extremity of the inner part of which goes to the bottom of the bottle. Above the cork a little tube, connected with a hand bellows, pierces the outer part of the double tube, and communicates by means of the outer part, by a small aperture, with the interior of the bottle. The inner tube for d elivering the ether runs upwards nearly to the extremity of the outer tube. Now, when the bellows are worked, a double current of air is produced, one current descending and pressing upon the ether forcing it along the inner tube, and the other ascending through the outer tube and playing upon the column of ether as it escapes through the fine jet. By having a series of jets to fit on the lower part of the inner tube, the volume of ether can be moderated at pleasure; and by having a double tube for the admission of air, and two pairs of hand bellows, the volume of ether and of air can be equally increased with pleasure, and with the production of a degree of cold six below zero.

"By this simple apparatus, at any temperature of the day and at any season, the surgeon has thus in his hands a means for producing cold even six degrees below zero; and by directing the spray upon a half-inch test-tube containing water he can produce a column of ice in two minutes at most. Further, by this modification of Siegle's apparatus he can distribute fluids in the form of spray into any of the cavities of the body—into the bladder, for instance, by means of a spray catheter, or into the uterus by an uterine spray catheter.

"When the ether spray thus produced is directed upon the outer skin, the skin is rendered insensible within a minute; but the effects do not end here. So soon as the skin is divided the ether begins to exert on the nervous filaments the double action of cold and etherization; so that the narcotism can be extended deeply to any desired extent. Pure rectified ether used in this manner is entirely negative; it causes no irritation, and may be applied to a deep wound, as I shall show, without any danger. I have applied it to the mucous membrane of my own eye, after first chilling the ball with the lid closed.

"I have now employed this mode of producing local anæsthesia in four cases on the human subject. The first case was the extraction of a tooth from a lady, the operation being performed by my friend and neighbor Dr. Sedgwick, on January 24th of this year. On the 29th of the same month, I used it again on the same lady for the extraction of three very difficult teeth, Dr. Sedgwick again operating. The results were as satisfactory as in the previous case, where the ice and salt ether apparatus was used.

"I have used the apparatus also in connection with my friend Mr. Adams, who had a case at the Great Northern Hospital of deep dissecting abscess in the thigh of a young woman. In the abscess there was a small opening, which just admitted the director. I first narcotized around this opening, and the director being introduced, Mr. Adams carried his bistoury nearly an inch deep and one inch in the line of the director. I then narcotized the deep-seated parts, and enabled him to cut for another inch and a-half in the same direction. The director was then placed in the upper line of the abscess, the process was repeated, and the incision was carried two and a-half inches in that direction. The patient was entirely unconscious of pain, and after narcotising the whole of the deep surface, Mr. Adams inserted his fingers and cleared out the wound without creating the slightest evidence of pain.

"Afterwards, in the case of a lacerated wound, six inches long, in the arm of a boy, who had been injured with machinery, I narcotized while six sutures were introduced by Mr. Adams. The first needle was carried through without the anæsthetic, and caused expression of acute pain; the remaining eleven needles, after a few seconds' administration of the ether spray, were passed through painlessly. The twisting of the wire sutures gave no pain.

"These results are so interesting that I make no apology for bringing them at once before my medical brethren. I wish it to be distinctly understood that at the present moment I only introduce the method here described for the production of superficial local anæsthesia. It is, I believe, applicable to a large number of minor operations, for which the more dangerous agent chloroform is now commonly employed—I mean such operations as tooth extraction, tying nævus, tying piles, incising carbuncles, opening abscesses, putting in sutures, removing small tumors, removing the toe-nail, dividing tendons, operating for fistula, removing cancer of the lip, and other similar minor operations which I need not mention. The process may also be applied to reduce local inflammation.

"In course of time, and guided by experience and the advancement of science, we may, however, expect more. If an anæsthetic fluid of negative qualities, as regards irritation of nerve, and which has a boiling point of 75° or 80° can be obtained from the hydrocarbon series, the deepest anæsthesia may be produced, and even a limb may be amputated by this method. It may also turn out that certain anæsthetics may be added to the ethereal solution with advantage, such as small quanties of chloroform, or some of the narcotic alkaloids, if they could be made soluble in ether. A solution of morphia and atropia combined, if they could be diffused through ether, which at present seems impossible, could thus be brought into action so as to cause deep insensibility. In operating on the extremities it would be good practice to stop the current of warm blood by making pressure above on the main artery.

"Reaction from the anæsthesia is in no degree painful, and hemorrhage is almost entirely controlled during the anæsthesia.

"One or two precautions are necessary. It is essential, in the first place, to use pure rectified ether; methylated ether causes irritation, and chloroform, unless largely diluted with ether—say one part in eight—does the same."—*Med. Times and Gazette*, Feb. 3, 1866.

